# Development of a health subindex for genetic selection of bulls and cows in Canadian dairy operations

**DOI:** 10.3168/jdsc.2025-0875

**Published:** 2025-12-01

**Authors:** Douglas W. Bjelland, John J. Crowley, Caeli M. Richardson, Natalie Howes, Peter R. Amer, Allison Fleming, Cindy Jaton, Christine F. Baes, Filippo Miglior

**Affiliations:** 1 AbacusBio, Dunedin 9016, New Zealand; 2 Lactanet, Guelph, ON, N1K 1E5, Canada; 3 Centre for Genetic Improvement of Livestock (CGIL), Department of Animal Biosciences, University of Guelph, Guelph, ON, N1G 2W1, Canada

## Abstract

This study presents the development of an updated health subindex (HSI) for genetic selection in Canadian Holstein dairy cattle, aimed at enhancing the genetic progress for health-related traits. The HSI was independently designed as part of a broader revision of the Lifetime Performance Index (LPI) and includes mastitis resistance (MR), metabolic disease resistance (MDR), fertility disorders (FD), hoof health (HH), and calf health (CH). The index was constructed by calculating trait-specific economic values, a transformation to relative breeding value units, and index testing using a dataset of 830 Holstein bulls with high genetic reliability. Economic weights were derived by integrating biological and economic data, including veterinarian examination costs, treatment costs, milk loss, and labor, and then adjusted for discounted genetic expressions to reflect trait expression timing and frequency. The final HSI showed moderate correlation with the previous health and fertility subindex (r = 0.61) and low correlation with other LPI components, indicating its distinct selection focus. The final percent emphasis of each trait in the HSI is 36.3% for MR, 31.9% for CH, 12.3% for MDR, 9.8% for FD, and 9.6% for HH. Selection response analysis revealed significant genetic gains in MR and CH when using the HSI for selection, demonstrating the subindex's potential to improve health outcomes in dairy herds. The HSI offers Canadian dairy producers a targeted tool for breeding healthier Holsteins, supported by updated economic and biological data and industry feedback.

## Highlights

• An updated health subindex was developed for Canadian dairy selection.
• The subindex was developed to be used to focus genetic selection for health traits.
• The greatest selection pressure is on improving mastitis resistance and calf health.

## Graphical Abstract

**Figure:**
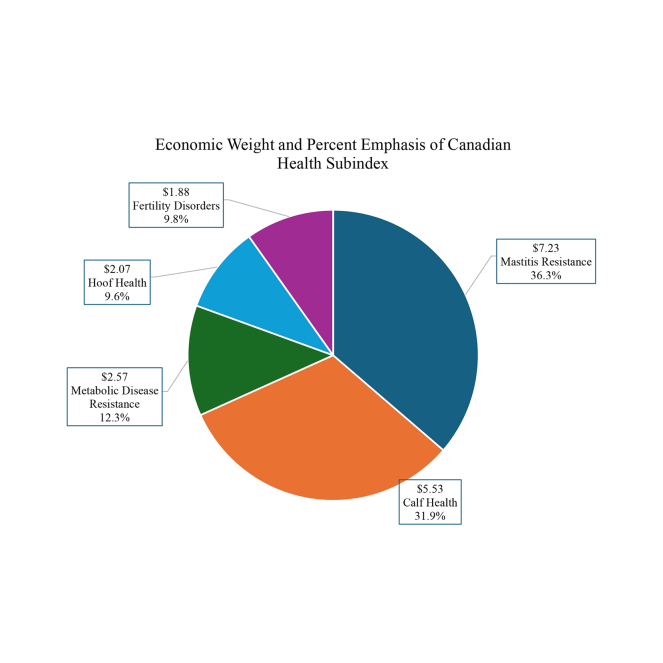
**Summary:** An updated health subindex has been developed for dairy cattle selection within Canada using current economic and biological factors. This subindex was developed to be used as a more focused view of the genetic superiority of health traits within bull and cow selection and to be used in conjunction with other subindexes (production, longevity and type, reproduction, milkability, and environmental impact) within the comprehensive Lifetime Performance Index. The economic weights, representing the change in value per 1-unit change in relative breeding value, ranged from Can$7.23 for mastitis resistance to Can$1.88 for fertility disorders. The finalized selection emphasis shows the greatest selection pressure on mastitis resistance at 36.3%, with the remaining selection emphasis on calf health (31.9%), metabolic disease resistance (12.3%), fertility disorders (9.8%), and hoof health (9.6%).

Selection indexes allow for the efficient selection of multiple traits simultaneously by accounting for economic and biological factors of each trait while also accounting for the genetic relationships among the traits (James, 1980). The economic aspect of selection indexes allow for the estimation of genetic superiority on a monetary scale reflecting the expected profitability to commercial farms. They are also an important decision-making tool for elite breeders and commercial producers aiming to direct their genetic selection toward specific market goals unique to their production system (Byrne et al., 2010). Along with economic factors, a desired gains approach can be considered and applied to indexes to ensure that the expected genetic response, selection emphasis, and economic weights (**EW**) are consistent with selection index end-users' expectations. Subindexes, often defined as a group of correlated or related traits from within a complete selection index (Zhang and Amer, 2021), can also be used to provide a more detailed perspective on an animal's genetic superiority in a specific genre of traits. Common subindexes include focusing on the selection of milk production (volume, fat, and protein production) within dairy cattle, growth traits (weaning weight, postwean gain, feedlot gain) for animals selected for meat production, fertility traits, or health traits (Berry and Amer, 2005; Sadeghi-Sefidmazgi et al., 2012; Zhang and Amer, 2021).

Comprehensive dairy selection indexes are commonplace throughout almost every major milk-producing country (Cole and VanRaden 2018). Relevant to the current work, health and longevity traits have been a focus within selection indexes in some countries for decades (Miglior et al., 2005; Heringstad and Østerås, 2013), whereas others have only recently included direct selection for health traits, previously relying on the correlated response from conformation or longevity traits (Sadeghi-Sefidmazgi et al., 2012; Parker Gaddis et al., 2014). In addition, Zoetis has included health traits within their commercial genomic tests and has been the first to include genomic evaluations for calf health traits (Zoetis, 2022).

In Canada, the Lifetime Performance Index (**LPI**) is a performance-based selection index made up of, until recently, 3 subindexes or components: production, durability, and health and fertility (Van Doormaal, 2024). The subindexes themselves hold traits that can also be a weighted sum (indexed) of other pertinent traits, for example, within the durability subindex, the hoof health (**HH**) “trait” includes 8 genetically evaluated traits: digital dermatitis, interdigital dermatitis, heel horn erosion, sole ulcer, toe ulcer, white line lesion, sole hemorrhage, and interdigital hyperplasia (Richardson et al., 2018). The Holstein health and fertility subindex includes daughter fertility (**DF**) and mastitis resistance (**MR**). In recent years, the Canadian dairy industry has been continuing to develop and investigate the potential for genetic evaluations of specific health traits, including MR (Beavers and Van Doormaal, 2013; Jamrozik et al., 2013), metabolic disease resistance (**MDR**; Jamrozik et al., 2016), fertility disorders (**FD**; Jamrozik et al., 2021), HH (Malchiodi et al., 2020), and calf health traits (Lynch et al., 2024). Incorporating these traits within a multitrait subindex, with a thorough calculation of the traits' economic impact to commercial producers, will enable robust selection leading to a more optimized genetic gain or, at the very least, allow for monitoring of selection responses and the maintenance of current genetic potential.

As part of a general reworking of the LPI, the objective of this study was to develop and test a new formulation of a health (only) subindex (**HSI**) for the Holstein breed. It was set out that the new HSI will include HH (% of daughters with no hoof lesion observed at trimming), MDR (% of healthy daughters with no clinical or subclinical metabolic disease during the transition period after calving), MR (% of healthy daughters for clinical mastitis), FD (% of healthy daughters with no fertility disorders), and calf health (**CH**; % of healthy calves). The HSI was developed in 3 main stages: economic value (**EV**) calculation per trait, transformation to relative breeding value (**RBV**) units, and index testing.

The calculation of trait-specific EV comprises assessing production costs and revenues related to changes in a particular trait by one unit (Amer et al., 1999). Extensive economic and biological data were used to calculate the EV in this first stage of development, with the economic inputs and solutions presented in Canadian dollars (all values listed as Canadian dollars; exchange rate of US$1.00 = Can$1.41). When Canadian-specific economic and production information was unavailable, data from broader North American dairy production systems were used, with global dairy information serving as a last resort. The calculations of EV for traits followed a general model that identifies and estimates costs per trait and considers their incidence to determine the total costs per trait (unit), such as

EV_trait_ = Vet_$_ + Labor_$_ + Treatment_$_ + Milk loss_$_ + Calf loss_$_,where Vet_$_ is the average veterinarian examination cost (examination cost multiplied by percent of cases requiring veterinarian examination), Labor_$_ is the average on-farm labor cost, Treatment_$_ is the treatment or antibiotic costs (or both) to treat a case, Milk loss_$_ is the cost of milk lost as a result of the of the treatment or disease (e.g., withholding milk with antibiotic residue, from decreased milk production, or a carryon effect of first lactation milk loss for the calf health traits), and Calf loss_$_ is the value of a calf that died or was culled due to calf diseases. These variables are presented in Table 1 with the resulting EV presented in Table 2.

**Table 1:** Calculations included in the economic values for disorders within the Holstein Health Subindex

Treatment	Mastitis	Ketosis	Subclinical ketosis	Displaced abomasum	Retained placenta	Metritis	Cystic ovaries	Diarrhea	Respiratory disease
Veterinarian examination cost^1^ (Can$)	21.44	23.08	0.00	381.83	12.08	54.37	60.41	21.44	23.08
Labor cost^2^(Can$)	1.69	9.66	0.00	43.47	9.66	9.66	2.42	1.69	9.66
Treatment cost (Can$)	36.00	35.00	0.00	382.00	20.00	105.00	27.60	143.25	59.28
Milk loss cost (Can$)	405.46	0.00	12.15	206.56	103.28	103.28	0.00	182.07^3^	109.46^3^
Calf loss cost (Can$)								27.00	16.20

1 Includes cost of veterinarian examination × percent of cases requiring veterinarian examination.

2 Includes hours of farm staff time required per case × Can$28.98 average hourly labor cost.

3 Includes expected first lactation milk loss given disease case.

**Table 2:** Calculation of economic weights from the cost per case and conversion factors

RBV	Trait	Economic value (cost/case, Can$)	Conversion factor^1^	Component economic weight^2^(Can$)	Index economic weight^3^(Can$)
Mastitis resistance		464.59	0.01556	7.23	7.23
Metabolic disease resistance					2.57
	Ketosis	67.74	0.00221	0.15	
	Displaced abomasum	1,013.86	0.00234	2.37	
	Subclinical ketosis	12.15	0.00425	0.05	
Fertility disorders					1.88
	Retained placenta	145.02	0.00328	0.48	
	Metritis	272.31	0.00367	1.00	
	Cystic ovaries	90.43	0.00444	0.40	
Calf health					5.53
	Diarrhea	352.32	0.00959	3.38	
	Respiratory	184.94	0.01165	2.15	
Hoof health^4^					2.07
	Toe ulcer	243.26	0.00125	0.30	
	Interdigital dermatitis	97.92	0.00227	0.22	
	Digital dermatitis	97.92	0.00816	0.80	
	Heel horn erosion	6.17	0.00324	0.02	
	Sole ulcer	67.42	0.00577	0.39	
	White line lesion	84.92	0.00321	0.27	
	Interdigital hyperplasia	12.92	0.00261	0.03	
	Sole hemorrhage	9.87	0.00352	0.03	

1 Conversion factor = change in percent of cases per RBV unit × desired gains factor × discounted genetic expression.

2 Component economic weight = economic value × conversion factor.

3 Index economic weight = sum of component economic weights.

4 Hoof health economic values previously derived by Richardson et al. (2018).

The average hourly labor cost (Can$28.98; CDC, 2023) was used in conjunction with the estimated farm staff time required to treat each disease to generate the farm labor cost. The average milk revenue per day of lactation (Can$20.66; CDC, 2023) was used to calculate estimated value of lost or reduced milk, along with the average number of days of reduced milk production before (10 d) and after (20 d) a clinical mastitis case (Sguizzato et al., 2024), the average percentage decline in milk production before and after a clinical mastitis case (25%; Sguizzato et al., 2024), length of subclinical ketosis cases (7 d), the average milk production loss during a subclinical ketosis event (8.4%), and the average number of days milk is withheld after treatments for mastitis, displaced abomasum, retained placenta, and metritis (6, 10, 5, and 5, respectively; Bruna Mion, University of Guelph, Guelph, ON, personal communication). Calf respiratory disease included the treatment costs of Can$59.28 (Dubrovsky et al., 2020) per disease instance per animal. The EV of calf diarrhea was calculated as a weighted average of mild (85% of total cases) and severe cases (15% of total cases) with the cost of treating severe cases (Can$120/d for 3 d) being significantly greater than the cost to treat mild cases (Can$35/d for 3 d; Roche et al., 2020). A loss in first lactation milk yield was also included as a cost of calf respiratory disease (121.2 kg; Buczinski et al., 2021) and calf diarrhea (126 kg per day of disease; Heinrichs and Heinrichs, 2011). Calves with respiratory disease or diarrhea were also estimated to have a 250% increased risk of calf death, culling, or removal (Windeyer et al., 2014; Buczinski et al., 2021) compared with an industry average of 2.4% (Winder et al., 2018). These cull and removal rates were combined with a female calf value of Can$450 (Quebec Financial Data, 2023) to determine the economic impact of losing a heifer calf.

The EV for hoof health traits were previously calculated by Richardson et al. (2018) and are presented in Table 2. These calculations incorporated data on digital dermatitis, heel horn erosion, sole hemorrhage, interdigital dermatitis, interdigital hyperplasia, toe ulcer, sole ulcer, and white line lesion, and included the cost of lesion treatments (by both farm staff and any required veterinarian assistance) and the labor costs from the time a treatment was initiated until the animal no longer exhibited clinical or subclinical symptoms. Given farmer acceptability and the fact that HH values are already published on the Lactanet platform, a decision was made use those values directly to maintain consistency.

Three factors (change in disease incidence per 1-unit RBV change, discounted genetic expressions, and desired gains factors) were used to transform the calculated EV into the final index EW. These 3 values, described in the following text, were multiplied together to create the conversion factors for each trait presented in Table 2.

Lactanet publishes the breeding values for traits relevant to HSI as standardized RBV (mean = 100, SD = 5, where a higher value is desirable). As the EV were calculated to reflect a 1-unit change in a trait, or a 1 percentage point change in incidence of a disease, a transformation is required to modify these values into a 1-point change in RBV. Conversion coefficients (EBV change per RBV change) were obtained from Lactanet, derived by a linear transformation using the EBV genetic SD and mean of the trait. These coefficients are proprietary information and cannot be published but are exampled by Fleming and Richardson (2021).

Using discounted geneflow methodology reported by Amer et al. (2001) and Berry et al. (2006), we calculated a total of 0.62 discounted cumulative genetic expressions of a bull's genes annually by lactating cows per calf born to the bull. Each lactation, a cow passes on one-half of these expressions to her calf, resulting in 0.31 discounted cumulative genetic expressions of a bull's genes annually by grand offspring via daughters of the bull for calf traits. However, the bull also passes on half of his genes to all calves born through direct service sire effects. Given this, there are 0.31 + 0.5 calf trait expressions for every 0.62 annual cow expressions, resulting in a ratio of 1.3 calf trait expressions per lactating cow trait expression. We incorporated this 1.3 discounted genetic expression factor in the presented conversion factor for the calf health traits to account for the more frequent and earlier expressions compared with the adult cow traits within the subindex.

While there are advantages from having a strictly economics-based index, consultation was undertaken with a panel of end-users and Canadian dairy industry experts to ensure the resulting relative emphases and selection responses would be best accepted by producers, genetics companies, breed associations, and other potential stakeholders utilizing the HSI. Slight desired gains modifications were included within the conversion factors listed in Table 2 to adjust the final subindex values to those most acceptable by the end-users. Several rounds of index testing, described in the following text, were conducted before finalizing the presented index values. The inclusion of the desired gains factors may limit the subindex's ability to maximize economic gain for producers; however, the anticipated increase in acceptance by stakeholders is expected to offset the potential economic inefficiencies.

The final EW presented in Table 2 indicate the effect on profitability per 1-unit change in RBV of each trait. The component EW is the product of the EV and conversion factor, with the index EW used for calculating the final subindex values being the sum of the component EW for each RBV. The EW values indicate that, for example, a 1-unit change in RBV for MR should yield a Can$7.23 change in profitability per cow per lactation. Given these EW, along with a sample set of animals with relevant EBV and RBV, index testing was performed on the developed subindex. The testing involved calculating the index on a sample of 830 Holstein bulls with an LPI reliability greater than 0.85 and born between 2015 and 2019. The next steps involved estimating and reviewing correlation between HSI and previous indexes, relative emphasis of traits, and trait responses over time given selection on the HSI. The bulls used within index testing were also required to have primary EBV for the in-development traits of calf diarrhea resistance and calf respiratory disease resistance.

The updated HSI has a correlation of 0.61 with the previous health and fertility subindex within LPI. The lower correlation is expected as the composition of the subindexes have changed with the removal of DF and the addition of MDR, HH, FD, and CH in the proposed HSI. The newly developed HSI has a low correlation (0.32) with the full LPI, as well as lower correlations with production (0.09) and durability (0.06) components of the LPI, indicating that the HSI is reflecting novel genetic selection compared with the other subindexes. The expectation is for HSI to have a percent emphasis of 8% within the updated full LPI, with the final value dependent on work outside the scope of this project. There are few favorable trait correlations, and several unfavorable correlations for the traits in the newly developed HSI with the production and durability subindexes. The herd life trait exhibits the greatest favorable correlation with all traits in the HSI, ranging from 0.19 (HH) to 0.47 (MR).

Relative emphasis of each trait in the HSI was calculated following the methods described by Zhang and Amer (2021). The majority of the selection emphasis is on MR (36.3%) and CH (31.9%), with lower emphasis on MDR (12.3%), FD (9.8%), and HH (9.6%). The large emphasis on MR is similar to what is seen in other health-specific indexes (Zoetis, 2022) and was viewed a necessity of the subindex by the advisory panel. A marginally higher proportion of the calf health EW within the HSI concentrated on the selection for calf respiratory disease, as compared with the focus observed in the Zoetis Calf Wellness Index (Zoetis, 2022). This is reflecting the desire by Canadian producers to focus more heavily on selection for improved respiratory disease resistance as this is currently seen as an issue on farms.

Responses in correlated traits were calculated by regressing the correlated trait value on the index value in the dataset of high LPI reliability bulls and scaling the resulting slope coefficients to result from a 1 SD change in HSI. The resulting response to selection (Figure 1) is an important aspect to understand the impact a selection index will have on the population and the values shown here represent the expected change in each breeding value (expressed as the proportion of a breeding value SD) from selecting bulls within the top SD of the developed HSI (blue) and the previous health and fertility subindex (orange). Given this is a subindex and not the singular selection focus, the results presented here are not reflective of what will materialize in the Canadian dairy herd, but it is still useful to provide evidence of the impact of the subindex in the population. The greatest selection response is seen in MR and SCS, as expected given the high emphasis placed on these traits. The other health traits included in the subindex, as well as correlated traits such as herd life, DF, and BCS are estimated to achieve significant favorable selection response if selection was solely performed using HSI, although the expected response is lower for both herd life and DF than in the previous health and fertility subindex. The inclusion of CH (shown within Figure 1 as the individual traits of calf diarrhea and calf respiratory disease) modifies the expected selection response from a neutral response seen in the previous health and fertility subindex to a significant positive response in the current update. The response for the FD traits of metritis, retained placenta, and cystic ovaries were also presented individually within Figure 1, with a slightly different response expected in the updated HSI.

**Figure 1:**
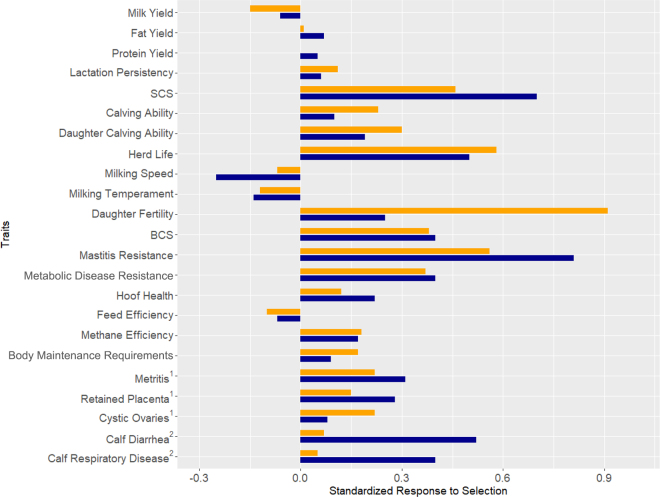
Standardized response to selection for all traits when selecting for the health subindex (blue) or previous health and fertility subindex (orange) for Holsteins. For all traits a positive correlation is more favorable. Responses are defined as the expected response to selection over a generation when selecting the top standard deviation of bulls for the Health Sub. ^1^Metritis, retained placenta, and cystic ovaries are individual traits within the RBV of fertility disorders (FD). ^2^Calf diarrhea and calf respiratory disease are individual traits within the RBV of calf health (CH).

The results of the updated HSI provide a focused view of the genetic superiority for selecting toward healthier Holstein animals within the Canadian dairy industry. Additionally, the inclusion of updated economic and biological inputs, along with industry-influenced adjustments, within the multitrait index provide up-to-date values to aid in genetic selection for Canadian dairy producers, and especially those producers interested in breeding for healthier dairy cattle.
